# Comparison of Neurite Density Measured by MRI and Histology after TBI

**DOI:** 10.1371/journal.pone.0063511

**Published:** 2013-05-22

**Authors:** Shiyang Wang, Michael Chopp, Mohammad-Reza Nazem-Zadeh, Guangliang Ding, Siamak P. Nejad-Davarani, Changsheng Qu, Mei Lu, Lian Li, Esmaeil Davoodi-Bojd, Jiani Hu, Qingjiang Li, Asim Mahmood, Quan Jiang

**Affiliations:** 1 Department of Neurology, Henry Ford Hospital, Detroit, Michigan, United States of America; 2 Department of Neurosurgery, Henry Ford Hospital, Detroit, Michigan, United States of America; 3 Department of Biostatistics and Research Epidemiology, Henry Ford Hospital, Detroit, Michigan, United States of America; 4 Department of Physics, Oakland University, Rochester, Michigan, United States of America; 5 Department of Radiation Oncology, University of Michigan, Ann Arbor, Michigan, United States of America; 6 Harper Hospital, MR Center, Detroit, Michigan, United States of America; University of Washington School of Medicine, United States of America

## Abstract

**Background:**

Functional recovery after brain injury in animals is improved by marrow stromal cells (MSC) which stimulate neurite reorganization. However, MRI measurement of neurite density changes after injury has not been performed. In this study, we investigate the feasibility of MRI measurement of neurite density in an animal model of traumatic brain injury (TBI) with and without MSC treatment.

**Methods:**

Fifteen male Wistar rats, were treated with saline (*n* = 6) or MSCs (*n* = 9) and were sacrificed at 6 weeks after controlled cortical impact (CCI). Healthy non-CCI rats (*n* = 5), were also employed. Ex-vivo MRI scans were performed two days after the rats were sacrificed. Multiple-shell hybrid diffusion imaging encoding scheme and spherical harmonic expansion of a two-compartment water diffusion displacement model were used to extract neurite related parameters. Bielshowski and Luxol Fast blue was used for staining axons and myelin, respectively. Modified Morris water maze and neurological severity score (mNSS) test were performed for functional evaluation. The treatment effects, the correlations between neurite densities measured by MRI and histology, and the correlations between MRI and functional variables were calculated by repeated measures analysis of variance, the regression correlation analysis tests, and spearman correlation coefficients.

**Results:**

Neurite densities exhibited a significant correlation (R^2^>0.80, p<1E−20) between MRI and immuno-histochemistry measurements with 95% lower bound of the intra-correlation coefficient (ICC) as 0.86. The conventional fractional anisotropy (FA) correlated moderately with histological neurite density (R^2^ = 0.59, P<1E−5) with 95% lower bound of ICC as 0.76. MRI data revealed increased neurite reorganization with MSC treatment compared with saline treatment, confirmed by histological data from the same animals. mNSS were significantly correlated with MRI neurite density in the hippocampus region.

**Conclusions:**

The present studies demonstrated that neurite density can be estimated by MRI after TBI and MRI measurement of neurite density is a sensitive marker to MSC treatment response.

## Introduction

Traumatic brain injury (TBI) is a major cause of mortality and disability, especially in children and young adults. Treatment has primarily focused on acute therapeutic intervention to reduce cellular damage and brain edema [1]. To date, there is no effective neuroprotective treatment to promote functional recovery after TBI [2], [3]. However, neurorestorative strategies designed to promote brain remodeling and to enhance functional recovery after various central nervous system (CNS) disorders, such as stroke, intracerebral hemorrhage, spinal cord injury, multiple sclerosis, and TBI, using pharmacological and cell based neurorestorative techniques have shown promising results in animals [4]. Treating brain injury with marrow stromal cells (MSCs) after stroke promotes axonal remodeling and increases oligodendrocyte formation [5], [6]. MSC treatment of TBI in rats significantly improves motor and sensory function measured using the modified neurological severity score (mNSS), and outcomes on learning and memory tests using the modified Morris water maze, compared to non-treated rats within days to weeks after treatment [7], [8], [9]. A recent report demonstrated beneficial outcome of autologous bone marrow mononuclear cell treated children with TBI [10].

Monitoring the progress of neuronal reorganization may permit determination of treatment efficacy after TBI. Current understanding of neuronal reorganization after brain injury has been primarily obtained from regional tissue measurements using histological and immunohistological methods which are restricted by single time point (terminal) analysis and therefore do not allow dynamic assessment of tissue remodeling. MRI offers excellent anatomical resolution, soft tissue specificity, and can be used for dynamic monitoring neuronal changes after TBI [11], [12]. Diffusion tensor imaging (DTI) has shown reduced fractional anisotropy (FA) in damaged areas of the brain during acute ischemic injury [13]. However, due to the assumption of Gaussian diffusion inherent to the tensor model, FA derived from conventional tensor analysis cannot resolve the fiber crossing problem [14], [15]. Reduced FA values in the area with crossing axonal bundles cannot be distinguished from true brain tissue axonal loss. The MR diffusion signal has a significant multimodal structure in clear disagreement with the conventional tensor model [15], [16], [17]. Solving the orientation distribution function (ODF), involves a complex set of q-space DWI (q-DWI) analysis [16], [17], [18], [19]. Employing the Diffusion Spectrum Imaging (DSI) has an advantage of extracting fiber information directly from the Fourier transform without applying any specific model, but it requires an extended data sampling time which may not be applicable for clinical data acquisition. The DSI method samples data points on dense Cartesian grids [14] and therefore requires a long acquisition time. A new approach using hybrid diffusion imaging (HYDI) requires fewer diffusion measurements thus shortening the scan time [20]. HYDI provides similar information as DSI by acquiring a set of combinations of multiple concentric shells [20]. Quantitative estimation of non-Gaussian water diffusion using the apparent kurtosis coefficient (AKC) has demonstrated its sensitivity for early stage axonal remodeling, which involves increased numbers of random crossing axons [18], [21], especially in external and internal capsule regions. However, AKC is not a biophysical measurement in direct response to a histological measurement, such as neurite density. Thus, there is a compelling need to provide a non-invasive biophysical measurement of neurites for quantitatively evaluating neurite reorganization. Models based on the non-Gaussian pattern have been proposed to estimate axon density, radius and slow compartmental water exchange membrane permeability in bovine optic nerves, sciatic nerves and mouse spinal cord [22], [23], [24]. The two-compartment model (i.e. “CHARMED” model) which considers water molecule diffusion patterns is either restricted intra-axonally or hindered extra-axonally [22]. However, diffusion gradients applied in “CHARMED” model have limitations for using perpendicular diffusion gradient to the fiber bundles which might not apply to general cases. Other q-space imaging methods to estimate microstructure of the whole brain without prior knowledge of the fiber orientation have been developed [25], [26], but these approaches have not been applied to evaluate neurite density distribution after neurological injury, such as TBI.

In the present study, we evaluate the effect of a restorative treatment of TBI using human marrow stromal cells (hMSC) [5], [6], [26] on neurite density measured by HYDI MRI [20] with spherical harmonic expansion of a two-compartment water diffusion displacement model [26] and histology after TBI. We also investigate the relationships between neurite density measured by MRI, histology and functional outcomes.

## Materials and Methods

### Animal Preparation

All experimental procedures were approved by the Institutional Animal Care and Use Committee of Henry Ford Health System.

Twenty male Wistar rats (weight:270–300 g, age:8–9 weeks) were divided into three groups, TBI+hMSCs treatment (hMSC treated group, n = 9); TBI+saline treatment (saline treated group, n = 6) and a normal group without neurological injuries (normal group, n = 5). All TBI rats were subjected to controlled cortical impact (CCI) [27]. For the induction of CCI, the head of each rat was mounted in a stereotaxic frame in a prone position and secured by ear bars and an incisor bar. Two 10 mm-diameter craniotomies were performed adjacent to the central suture, midway between the lambda and the bregma, leaving the dura matter over the cortex intact. The left craniotomy confined the location of experimental impact while the right one allowed for the lateral movement of cortical tissue. Injury was induced by a pneumatic impact device [27] on the intact dura. A single strike was delivered at 4 m/sec with a 2.5 mm of compression to the left cortex with a pneumatic piston containing a 6-mm-diameter tip [7], [8], [9]. After the impact, the bone plate was not replaced and was sealed with bone wax, the skin was then sutured with 4-0 surgical thread. Human MSCs (hMSCs) were provided by Theradigm (Baltimore, MD) and were prepared using the same procedures as described in our previous study [21]. The TBI animals either received 3×10^6^ hMSCs (hMSC treated group) in a 2 ml volume or saline (saline treated group) intravenously (IV) injected 6 days after TBI. All TBI rats were sacrificed at 6 weeks after CCI.

### MRI Neurite Density Measurement

To evaluate neurite density in brain without prior knowledge of specific fiber orientations, the composite hindered and cylindrical diffusion model [26], and hybrid multiple concentric shell data acquisition scheme [20] were used in the present study. The MRI model of neurite density proposed by Jespersen [26], [28] was employed. MR signal is assumed to originate from two non-exchanging components, one with cylindrical symmetry, describing diffusion in neurites (dendrites and axons), and one with hindered components, accounting for diffusion in the remaining compartments, among which isotropic water diffusion was assumed [26], [28]. Axonal fiber information can be extracted from principal diffusion directions [26], [28]. Due to the minor contribution of higher order of spherical harmonics, the signal equation was truncated at lower order (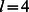) level in spherical harmonic forms. Neurite density maps were generated on a voxel-by-voxel basis using an in house optimization fitting program written in Matlab. Orientation distribution function (ODF) and probability distribution function (PDF) were calculated by extracting over-determined matrix for least square solutions [20].

### MRI Data Acquisition

Ex-vivo animal brain data acquisition was performed two days after sacrifice on a Varian 7 Tesla, 20 cm bore superconducting magnet (Palo Alto, CA) system with gradient up to 290 mT/m and a 38 mm quadrature coil. Multiple shell q-space DWI using HYDI data acquisition scheme [20] was performed using a conventional spin echo readout with 24 mm FOV, 128×128 imaging matrix, 1 mm slice thickness with 13 slices, TR/TE = 2500/40 ms, δ = 10 ms, Δ = 18 ms, 125 uniformly distributed diffusion attenuated directions with b-values of 0, 360, 1440, 3240, 5760 and 9000 s/mm^2^ in each slice. The SE readout in current HYDI DTI avoids the image distortion compared with echo planar imaging (EPI), but it increased total acquisition time up to approximately 27 hours with NEX = 5. T_2_ measurement was performed using standard two-dimensional Fourier transform (2DFT) multi-slice multi-echo MRI. T_2_ maps were obtained using TEs of 20, 40, 60, 80, 100 and 120 ms and TR = 3000 ms, with the same FOV and matrix size and slice number as HYDI images. The duration of the entire T_2_ sequence was approximately 3.2 mins. Normal rats were sacrificed and then followed the same procedures and measurements as TBI rats.

### Histological Staining

#### Tissue preparation and Bielshowski Luxol Fast Blue staining

TBI rats were sacrificed at 6 weeks after TBI. The procedures to prepare brain sections were the same as previously reported [21]. Bielshowski and Luxol fast blue staining [29] were used to identify reticular fibers (i.e. neurofibrils and neurofibrillary tangles) and myelin, respectively. For immuno-histochemical staining, slides were placed in 20% silver nitrate in the dark, and ammonium hydroxide was added until the tissues turned brown with a gold background, and then sodium thiosulfate was added [30]. The slides were then stained for Luxol fast blue, washed in 95% alcohol and placed in lithium carbonate. Nuclei are colorless; myelin is blue and axons are black.

### Data Analysis

#### Histological analysis

**Figure 1:**
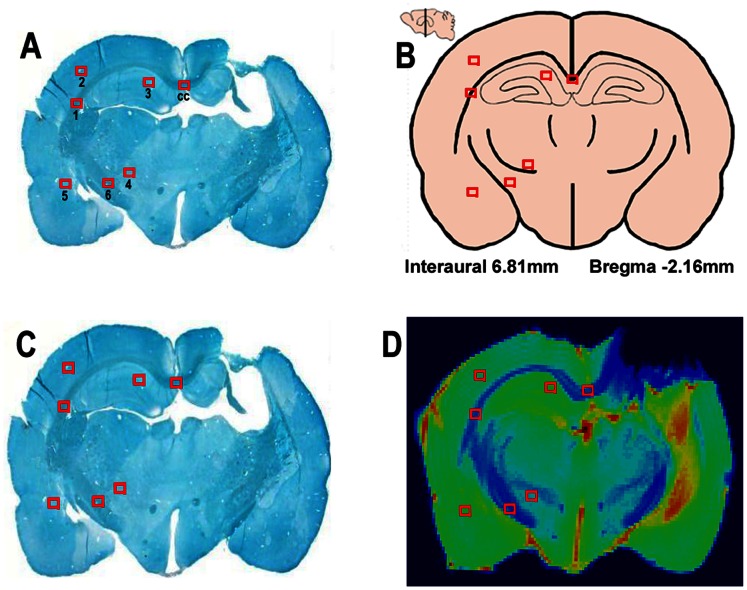
Locations of selected ROIs in original Bielshowski Luxol Fast Blue staining section (A), anatomic brain structures (B) [31], warped staining section (C) to T2 weighted image and corresponding warped ROIs in the T2 weighted image (D).

**Figure 2:**
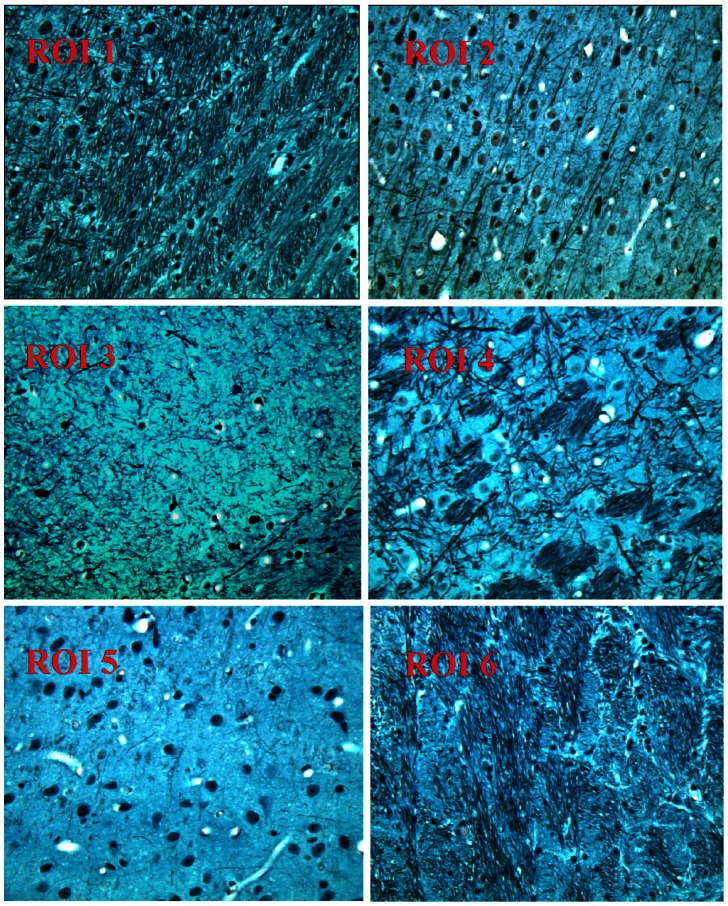
Histological details in selected ROIs in contralateral side of the brain. ROI 1: external capsule; ROI 2: prim somatosensory cortex; ROI 3: hippocampus; ROI 4: internal capsule; ROI 5: insular cortex; ROI 6: striatum were listed from up left (ROI 1) to bottom right (ROI 6) [31].

ROIs were selected based on different brain anatomical regions [31]. A total of thirteen ROIs were selected on the E-slice section (bregma 1.56–3.56, Figure 1) [31] with six ROIs (Figure 2) on each hemisphere and the corpus callosum ROI in the center. The six ROIs include external capsule (ROI #1), primary somatosensory cortex (ROI #2), hippocampus (ROI #3), internal capsule (ROI #4), insular cortex (ROI #5), and caudate putamen (striatum) (ROI #6) and corpus callosum (ROL #CC). Similarly, we analyzed a total of 12 ROIs on the F-slice section (bregma 3.56–5.56) [31] including the visual cortex (ROI #1), mesencephalic reticular formation (ROI #2), periaqueductal gray (ROI #3), red nuclus, parvicellular part (ROI #4), cerebral peduncle (ROI #5), part of the substantia nigra medial lemniscus (ROI #6) [31]. ROIs encounter both the lesion and the contra-lateral hemisphere of the same rat brain (Figures 1 and 2). Light microscopy images (40× magnification) were obtained from the specific ROIs (375×375 µm^2^). Histological axonal density data was calculated using ImageJ software (Version 1.42q) [32], [33] with a threshold based on the maximum entropy value of all major identifiable axons having minimum overlap with adjacent major axons. Dark soma regions were discarded from the images. The threshold was slightly adjusted according to the microscopic image slice intensity (threshold value +/−10). Volume fraction was considered as neurite density for each selected ROI. A detailed histological analysis method is presented in our previous study [33].

#### Image processing

MRI data were processed using Matlab (MathWorks, Natick, MA). Because histological sections are more distorted than the T_2_-maps, the histopathology sections were warped to fit the corresponding T_2_ images (Figure 1). A non-rigid transformation [34], [35] and a B-spline grid based manual warpping methods [36] were used for registration of the histology image to T_2_ weighted images. ROIs locations were saved on T_2_ maps (Figure 1D) and applied to all the calculated MRI maps. FA maps were created using b = 1440 s/mm^2^ data using DTIStuido [37]. To evaluated the changes in the TBI boundary between hMSCs treated and saline treated group animals, five evenly separated TBI boundary ROIs (2×2 pixels) were chosen from the extended boundary band (5 pixels) of the core lesion region on T_2_ maps. Boundary ROIs were saved and applied to MRI parameters and neurite density. The injured lesion core was identified on the T_2_ maps using the threshold T_2_ value of mean±2 standard deviations of the T_2_ values in the contra-lateral hemisphere [21].

### Functional Outcome

TBI animal functional outcome was evaluated using the modified neurological severity score (mNSS) [38] and the modified Morris water maze test [9]. Functional outcome was compared with MRI neurite density and histology. The mNSS grades the composite neurological function of an animal on motor, sensory, reflex and balance tests. One point is given for the inability of an animal to perform the tasks correctly or for the lack of a tested reflex. The higher the mNSS score the more severe neurological dysfunction. mNSS was assessed for each animal pre-TBI and post-TBI on days 1 and weekly thereafter by an examiner blinded to the treatment groups and the corresponding MRI results. Modified Morris water maze test (WMT) was performed during the last 5 days before being euthanized to evaluate the long-term functional outcome of spatial learning acquisition and memory retention [9].

### Statistics

To study the correlation of neurite density measured between histology and MRI, a linear clustered regression model was used by accounting for the multiple ROIs, slides and different treatment per subjects. Regression analysis tests the significant coefficient of MRI with the estimation of R^2^ for model goodness-of-fit, where the correlation coefficient is 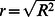. Intra-correlation coefficient was calculated to assess the agreement (1∶1 match) of the two measures. Correlation of histological and MRI neurite density and FA on the lesion side of three groups (hMSC/saline treated or normal group) were performed by using the same analysis approaches as described above. Repeated measurement analysis of covariance (ANCOVA) was used to study the group difference adjusting multiple regions per subject. The analysis started testing for the overall region and group effect, followed by the subgroup analysis of the group difference (cell treated vs. saline treated, or saline treated vs. normal) at each region. Two-sample t-test was used to test the treatment effect for WMT and the Wilcoxon test was used for mNSS. Spearman correlation coefficients were calculated between MRI measurements in each ROI and functional variables, adjusting for the study treatments. Functional behavioral data in the correlation analysis used mNSS at 35 day and average WMT from day 30 to 35 as variables.

Two groups of TBI animals, hMSC treated or saline treated, were used to test treatment effects on neurite density and FA measured from the 5 additional ROIs of the extended TBI lesion boundary. Repeated measurement analysis of covariance (ANCOVA) was used to study the treatment effect. Subgroup analysis used restricted maximum likelihood (REML) estimation in R, a mixed procedure for each ROI in different animal groups.

## Results

### Correlation between MRI and Histology

**Figure 3:**
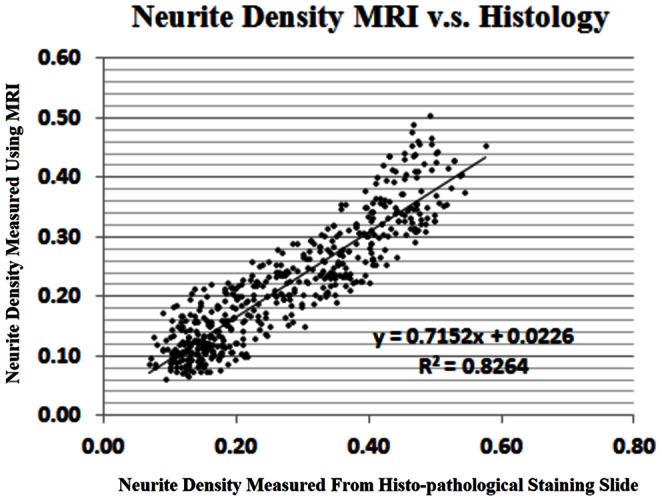
Comparison of neurite density measured by histology and MRI. Significant correlations were detected between histological and MRI neurite densities.

The MRI neurite density map visually matches well with the corresponding Bielshowski Luxol Fast Blue staining section in the anatomical regions, where the corpus callosum (ROI cc) and striatum (ROI 6) showed higher density than other selected ROIs. Significant correlations between MRI and histological neurite density were detected in TBI left (R^2^ = 0.83, P = 0.006), right (lesion side, R^2^ = 0.82, P = 0.001), normal left (R^2^ = 0.87, P = 0.6), normal right (R^2^ = 0.86, P = 0.06). All three group ROI data sets were used to determine the overall correlation, and a significant correlation was observed as shown in Figure 3. (R^2^ = 0.83, P = 0.0001). FA values correlate moderately with histological staining results with R^2^ = 0.59, P<E-5. Intra-correlation coefficient also exhibited excellent agreement between histological and MRI neurite density (95% lower bound as 0.86) but weaker agreement between histological neurite density and FA (95% lower bound as 0.76).

### Quantitative Evaluation of hMSC Treatment Effectiveness

**Figure 4:**
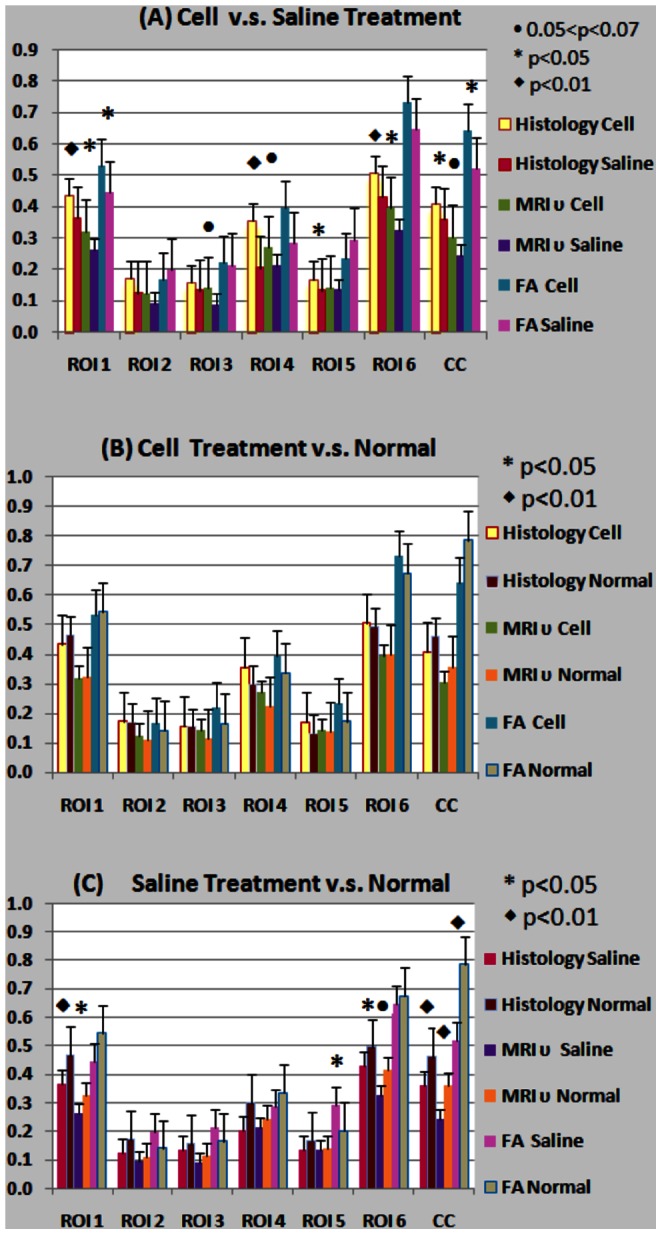
Direct comparison between histological and MRI neurite densities and FA between hMSCs treatment (n = 9) and Saline-treatment groups (n = 6) (A), hMSCs treatment and normal healthy groups(n = 5) (B), and Saline treatment and normal healthy groups in TBI lesion side ROIs.

Figure 4 shows the direct comparisons of histological and MRI neurite density and FA in 7 ROIs of the lesion hemisphere in section E between hMSC treated and saline treated rats (Figure 4A), and between hMSC treated and normal healthy rats (Figure 4B), and between saline treated and normal healthy conditions (Figure 4C). ANCOVA analysis of overall group and ROI effects showed significant differences between hMSC treated vs. saline treated (Pr> |t|, <0.0001, Pr: probability) and saline treated vs. normal animals (Pr>|t|, <0.0001). Also significant differences were present in neurite density (υ) and FA values between selected ROIs (Pr>F, <0.0001) in all three animal groups.

Subgroup analysis results were organized and compared by measurement methods in a total of seven selected ROI’s in the TBI lesion side of the brain. There are significant overall group differences in histological neurite density and MRI neurite density measurements among the three subgroups. No overall significant difference in FA was found among the three subgroups. Statistical significance among subgroups was listed in Table 1. Figure 4(A) shows the MRI measured neurite density of the hMSC treated group was significantly higher in the external capsule (ROI 1), somatosensory cortex (ROI 2), and in the striatum (ROI 6). The MRI neurite density of the hMSC treated group was marginally higher in the hippocampus (ROI 3), internal capsule (ROI 4) and corpus callosum (ROI cc) when compared to the same ROI in saline treated animals. Neurite density data measured by histology showed similar significant changes between cell treated and saline groups, with slightly lower p-values (Table 1). In Figure 4(B), hMSC treated TBI animals had no significant differences in neurite density compared with healthy normal animals. FA and neurite density exhibited lower mean values in the external capsule (ROI 1) and in the corpus callosum (ROI cc) regions of hMSC treated TBI animals when compared to the normal group. However, the hMSC treated TBI group showed a slight increase the mean values in FA and neurite density in ROIs 2–6. Saline treated TBI animals in Figure 4(C) showed a decrease in neurite density in all selected ROI’s compared to those ROIs in the normal group, with significant decreases in ROI 1, 6 and ROI cc. Slight decreases in other ROIs are evident in sub-cortical regions and are indirectly influenced by CCI. Significantly decreased FA values (p<0.01) in the saline treated group present in the corpus callosum (Figure 4(C) ROI cc) were in agreement with histological and MRI measured neurite density. This is primarily caused by fiber track damage due to the traumatic impact. FA values were inconsistent with histological and MRI neurite density values in somatosensory, hippocampus and insular cortex, as shown in the last two sub-columns in ROI 2, 3, 5 in Figure 4C.

**Figure 5:**
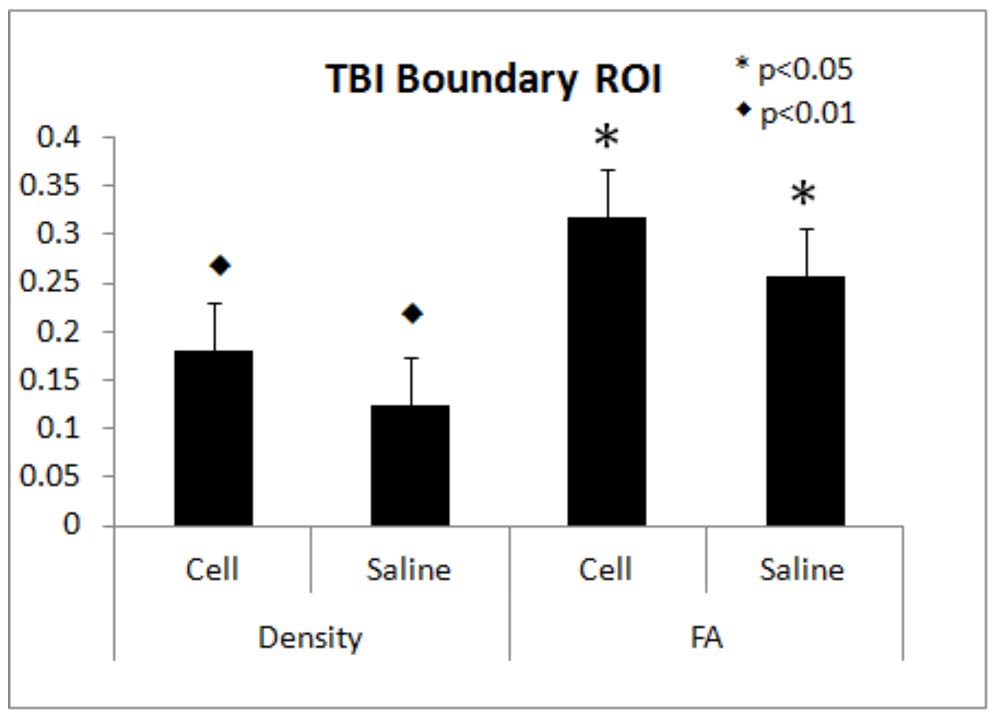
Neurite Density and FA values (%) from extended TBI boundary ROI under cell (n = 9) and saline treated (n = 6) conditions. Significant increase in MRI neurite density and FA (p<0.01) were detected in hMSC treated compared with saline treated group animals, *p<0.05 and ♦ p<0.01.

In addition, neurite density around the TBI boundary regions between the cell and saline treated animals were also compared to evaluate treatment effects on neurite remodeling in TBI boundary areas. Significant increase in neurite density (p = 3E−4) and a marginal effect on FA (p = 0.045) were present in hMSC treated animals (Fig. 5).

### Functional Outcome

**Figure 6:**
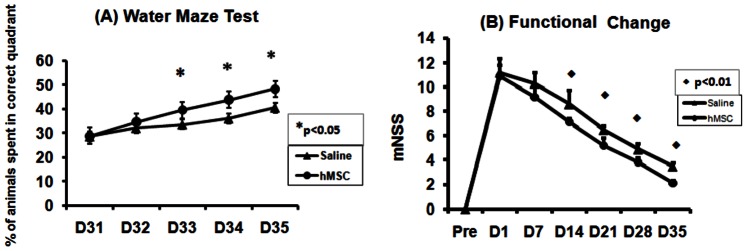
Functional recovery after TBI. Significant functional improvement was detected by the Morris water maze test (A) and modified neurological severity score (mNSS) (B) in hMSC treated group (n = 6) compared with saline treated groups (n = 6), *p<0.05 and ♦ p<0.01.

Improved functional performance in hMSC treated TBI animals was observed in both water maze test and mNSS scores. Cell-treated animals exhibited significantly improved Morris Water Maze test compared with saline-treated animals starting from day 33 (p<0.05, Figure 6A). Although both hMSC-treated and saline-treated TBI animals showed decreased functional mNSS scores with time, hMSC treated group exhibited lower (p<0.01) mNSS scores than that in saline-treated group from day 14 after TBI (Figure 6B). mNSS was also significantly correlated (r = 0.64, p = 0.035) with MRI neurite density in the hippocampus region (ROI 3). FA values showed no correlation with functional tests.

## Discussion

Although previous investigations exhibited promising results in detecting axonal reorganization using kurtosis, FA, and q-ball images [21], [39], there have been no investigations to measure histological related MRI neurite density after TBI with and without a neurorestorative treatment, such as hMSCs. In the current study, a two-compartment model [26] without prior knowledge of the fiber orientations and the HYDI data sampling scheme [20] was employed to compare MRI neurite density with corresponding immuno-histopathological measurements in normal animals and animals with and without hMSC treatment after TBI. We demonstrated that MRI neurite density is highly correlated with corresponding histological measurement under both TBI with and without hMSC treatment and under non-TBI conditions. MRI neurite density also exhibited better agreement with histological neurite density measured by intra-correlation coefficient compared with FA. As discussed in the previous section, the cell treated group showed improved functional recovery after TBI, which corresponds to increased neurite density (Figure 4A, Figure 5) and FA in both TBI boundary and specific anatomical regions. These data demonstrate for the first time that MRI measurement of neurite density is an important imaging marker for neurite reorganization after brain injury and possibly for functional recovery.

High agreement between histology and MRI neurite density (95% lower bound as 0.86), indicates MRI is applicable for noninvasively measuring neurite density in brain. The value ranges of our MRI neurite density and FA are comparable with a previous report [28] in all corresponding ROIs. However, we investigated the feasibility of MRI neurite density in detecting neurite reorganization after TBI and confirmed our approach by histological measurements with fewer gradient directions and a more uniformly distributed gradient sampling scheme. Imaging white matter (WM) injury or recovery after TBI had been performed predominately using traditional DTI measurement, such FA and fiber tracking. Consistent with our results, MacDonald [39] found significant correlation between FA and axonal density in the ROI with one direction axonal bundles. Although traditional DTI is promising in evaluating WM injury, it cannot detect axonal reorganization with random crossing axonal bundles, especially during the early stage of neurite reorganization after brain injury [21]. This may be one reason for the relatively lower agreement of FA than MRI neurite density with the histologically measured neurite density and our relative low correlation coefficient between FA and histological neurite density compared with Dr. MacDonald’s study. To overcome the weakness of traditional DTI, different quantitative q-space DTI measurements, such as kurtosis have been applied to quantify axonal reorganization after brain injury. The apparent kurtosis coefficient map exhibits increased AKC but reveals low FA in the TBI boundary with random oriented axons, which was confirmed by the q-ball fiber orientation map and high magnification Bielshowski and Luxol fast blue staining images [21]. Although kurtosis measurement has advantages in detecting axonal reorganization with random oriented axons, it is not a biophysical measurement directly in response to the histological measurements of neurite density. Our data demonstrate that the MRI neurite density measurement used in the current study is in agreement with histological measurement of neurite density and could dynamically monitor neurite changes in animals and even patients due to the non-invasive nature of MRI. Our data indicate that MRI neurite density can detect treatment effects after TBI in external/internal capsules and striatum as well as the TBI boundary. Increased neurite density in the injured boundary with improved functional recovery after neurorestorative treatment in current study agrees with previous investigations [13], [21], [40].

In this study, we observed increased neurite density of hMSCs treated group animals. Reasons for increased neurite density and reorganization induced by hMSC treatment may be related to the production and parenchymal cell stimulation of neurotrophic factors by hMSCs [41] and increased tissue plasminogen activator (tPA) activity in astrocytes which drives axonal remodeling in hMSC treated stroke animals [5], [42]. Neurorestorative treatment increases both progenitor and mature oligodendrocytes in the ipsilateral hemisphere of the injured brain [5]. Oligodendrocytes generate myelin and contribute to the integrity of white matter tracks in the brain. Stimulation and amplification of these oligodendrocytes by hMSCs may lead to restructuring of axons and myelin. White matter architecture in the injured boundary is altered by neurorestorative treatment, and axonal density in the periinfarct area is significantly increased in the treated animals [5], [21]. Pseudorabies virus labeled with green fluorescent protein (PRV-GFP) and red fluorescent protein (PRV-RFP) has been used to demonstrate axonal remodeling in an experimental brain injury animal model with spontaneous recovery and after restorative cell therapy [43]. The orientation of axonal bundles during early stage of neuronal reorganization is somewhat random [21]. hMSC treatment induced neurite remodeling in the injured TBI lesion boundary, as previously reported [21], was also detected in selected cortical and subcortical regions (EC, IC and striatum) in the current study. The cell-based treatment may also reduce the dieback of axonal bundles compared with the saline treatment. Therefore, the hMSC treatment may also benefit these tissues by increasing axonal density after TBI as has been reported after stroke [44].

Functional mNSS neurological test results show significant correlations with MRI neurite density measured in ROI 3 (r = 0.64, p = 0.035). From Figure 1 we can see that ROI 3 is the ROI nearest to TBI lesion. The axonal bundles in the TBI boundary reorganize and may reestablish connectivity for the lost functions from the TBI damage. Axonal reorganization is primarily localized in the TBI boundary, close to lesion, as demonstrated in the current and previous investigations [26]. The axonal reorganization in the ROI near the TBI lesion may indicate an improved functional score. The TBI damaged areas are mainly located in motor and sensory cortex. The significant neurite density difference between hMSC treated and saline treated group animals in these lesion regions are detected by MRI neurite density measurement, and are significantly correlated to the outcome measured by the mNSS functional test. The remodeled tissue structures, as presented by increased MRI neurite density, may promote the reestablishment of axonal connections to improve functional behavior [13], [21].

Further improvements of the combination of HYDI data acquisition with the neurite density modeling [28] may arise from two aspects: experimental setup and the diffusion model itself. Experimental improvements may require fast data acquisition with advanced pulse sequences, increased multi-channels for head coil, partial k-space data acquisition and sensitivity encoding (SENSE). The diffusion model used in the current experimental setup is sensitive in detecting myelinated axons. Even though it can capture some dendrites, the majority of dendritic contributions are underestimated [28]. The diffusion model used in this study is a two-compartment model which contains extra-cellular isotropic diffusion and intra-cellular diffusion within the cylindrical compartments. This model does not include water exchange between cylindrical and extra-cellular compartments. A more realistic diffusion model should include the compartmental water exchange and cylinder radii effects of diffusion signal contribution in different brain anatomical regions. Thus, a neurite radii weighting factor [28] needs to be included in future diffusion models.

### Conclusion

Neurite density is a valuable marker directly related to the connectivity of key nervous system pathways. Functional recovery is related to neurite structure reorganization. To date, no MRI measurement of neurite density changes after neurological injury, such as TBI with or without treatment has been performed. Here we demonstrated for the first time, that the MRI measurement of neurite density after TBI is highly consistent with histologically measured neurite density and is sensitive to treatment effects. MRI neurite measurement could potentially be used in clinical patient study due to its non-invasive nature and may be applicable to many other neurological diseases, such as stroke, spinal cord injury, multiple sclerosis, and aging related neuro-degenerative diseases.
